# The Structural Diversity of Marine Microbial Secondary Metabolites Based on Co-Culture Strategy: 2009–2019

**DOI:** 10.3390/md18090449

**Published:** 2020-08-27

**Authors:** Jianwei Chen, Panqiao Zhang, Xinyi Ye, Bin Wei, Mahmoud Emam, Huawei Zhang, Hong Wang

**Affiliations:** 1 College of Pharmaceutical Science & Collaborative Innovation Center of Yangtze River Delta Region Green Pharmaceuticals, Zhejiang University of Technology, Hangzhou 310014, China; cjw983617@zjut.edu.cn (J.C.); 18668358826@163.com (P.Z.); xinyiye1020@zjut.edu.cn (X.Y.); binwei@zjut.edu.cn (B.W.); mahmoudemamhegazy2020@gmail.com (M.E.); hwzhang@zjut.edu.cn (H.Z.); 2 Phytochemistry and Plant Systematics Department, National Research Centre, 33 El Bohouth St., Dokki, Giza 12622, Egypt

**Keywords:** co-culture, marine microbes, natural products, structural diversity, biological activities

## Abstract

Marine microorganisms have drawn great attention as novel bioactive natural product sources, particularly in the drug discovery area. Using different strategies, marine microbes have the ability to produce a wide variety of molecules. One of these strategies is the co-culturing of marine microbes; if two or more microorganisms are aseptically cultured together in a solid or liquid medium in a certain environment, their competition or synergetic relationship can activate the silent biosynthetic genes to produce cryptic natural products which do not exist in monocultures of the partner microbes. In recent years, the co-cultivation strategy of marine microbes has made more novel natural products with various biological activities. This review focuses on the significant and excellent examples covering sources, types, structures and bioactivities of secondary metabolites based on co-cultures of marine-derived microorganisms from 2009 to 2019. A detailed discussion on future prospects and current challenges in the field of co-culture is also provided on behalf of the authors’ own views of development tendencies.

## 1. Introduction

**Figure 1:**
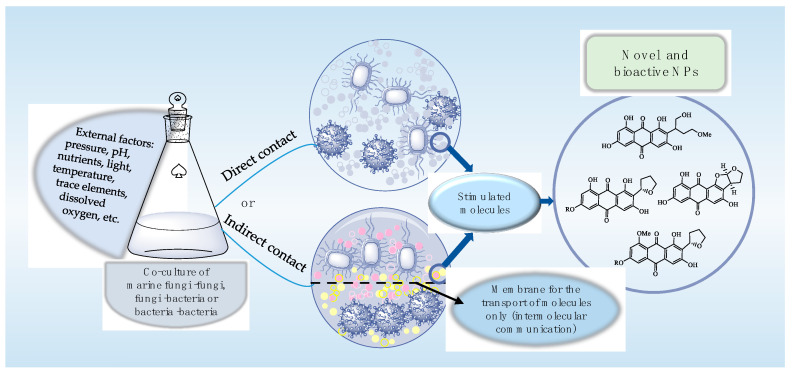
The schematic diagram of novel and bioactive natural products (NPs) using co-cultures of marine fungi−fungi, fungi−bacteria and bacteria−bacteria in direct or indirect contact.

Although many industrial sectors have stopped their dependence on natural product (NP) drug discovery programs, NPs are still of great interest to many pharmaceutical communities and are important sources of bioactive compounds [1,2]. Marine microbes, as an important source of bioactive NPs, have elicited widespread attention [3,4,5]. However, the discovery of novel marine microbial NPs is becoming more difficult and the rate of rediscovery of known NPs is being gradually increased. On the other hand, recent genomic sequencing has revealed the presence of numerous biosynthetic gene clusters in some microbes that may be responsible for the biosynthesis of NPs which are not found under classical cultivation conditions [6,7]. Therefore, many alternative strategies have been explored to activate these silent and cryptic biosynthetic genes. The co-culturing of marine microbes involves the culturing of two or more marine microbes together on/in certain conditions; microorganisms can communicate with each other through direct or indirect contact, thereby stimulating the silent gene clusters to produce special NPs [2,8] (Figure 1). This strategy can promote the production of complex and novel skeletons with numerous stereocenters [9,10,11]. Hence, the co-culturing of marine microbes draws widespread attention in the scientific community as a potential source of unknown bioactive substances classified as alkaloids, polyketides, anthraquinone, flavonoids, cyclopeptides, etc. To exploit the NPs from the co-cultures of marine microbes and understand their medicinal significance, this review summarizes successful examples involved in NPs of marine microbes based on co-cultures from 2009 to 2019 (Table 1).

## 2. Compounds Derived from the Co-Cultures of Marine Microorganisms

**Figure 2:**
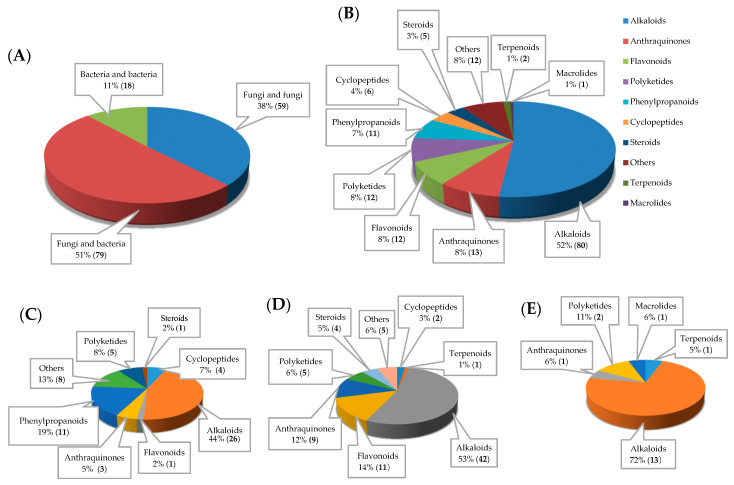
Numbers and the percentage of (**A**) isolates from the co-cultures of different marine microbes; (**B**) different classes of NPs from the co-cultures of marine microbes. The classes, numbers and proportions of NPs isolated from the co-cultures of marine (**C**) fungi and fungi, (**D**) fungi and bacteria, (**E**) bacteria and bacteria.

Co-culturing or mixed fermentation is considered an important technique of inducing secondary metabolites hidden in the genomes of marine microbes by using appropriate physiological conditions, chemical communication and competition of microbes. Consequently, it is considered an easy, cheap and effective method [12,13]. This finding also explains the chemical communication and antagonism between different marine microorganisms, such as the interactions between marine fungi−fungi, fungi−bacteria and bacteria−bacteria, in which they act as signaling molecules, competitors or defense agents [14]. Herein, the metabolites based on co-cultures of marine microbes were classified according to their skeletons as alkaloids, anthraquinones, cyclopeptides, flavonoids, macrolides, phenylpropanoids, polyketides, steroids, terpenoids and others from 2009–2019. These excellent examples were found from SciFinder, Science Direct, PubMed, Springer and other databases. Among them, the interactions between marine fungi and bacteria were found to induce the most metabolites (Figure 2A), and the alkaloids played a significant role in co-cultures of marine microbes (Figure 2B), no matter whether the mixed cultivation was of marine fungi−fungi (Figure 2C), fungi−bacteria (Figure 2D) or bacteria−bacteria (Figure 2E).

### 2.1. Alkaloids

**Figure 3:**
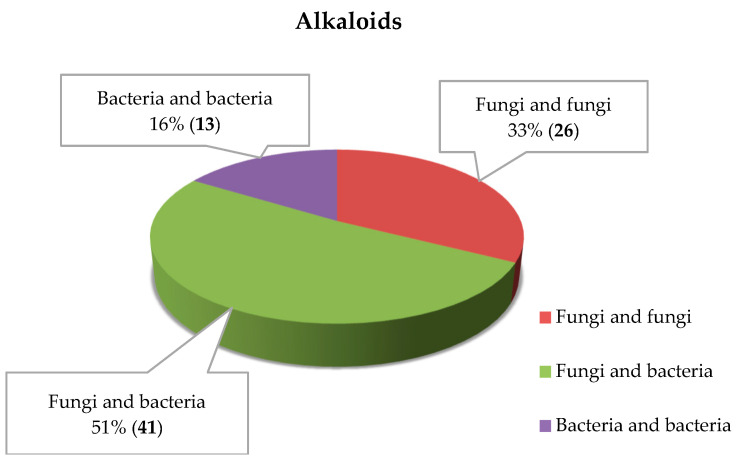
Alkaloids isolated from the co-cultures of marine fungi–fungi, fungi–bacteria and bacteria–bacteria.

The nitrogenous alkaloids represented the most abundant class of compounds that were produced by the co-cultures of marine microorganisms with diverse skeletons and biological activities [15,16]. Eighty alkaloidal metabolites were isolated and identified from different microbial environments (Figure 2B), and the co-cultures of marine fungi–bacteria represented 51% of the total isolates (Figure 3).

#### 2.1.1. Alkaloids Derived from the Co-Cultures of Different Marine Fungi

**Figure 4:**
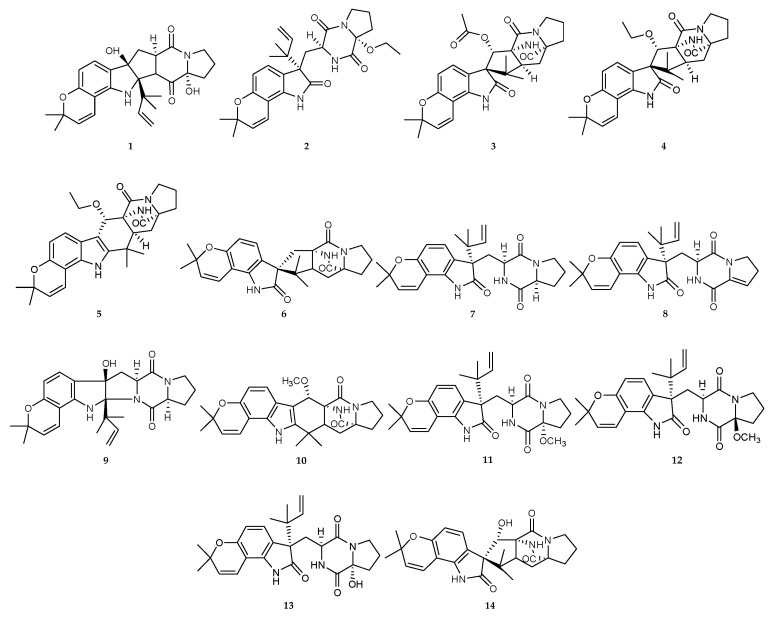
Chemical structures of **1**–**14**.

Several studies of co-cultures of fungal–fungal interactions from different marine sources were summarized as follows; 26 alkaloids were isolated and identified (Figure 2C and Figure 3). The mixed fermentation of marine-derived fungi *Aspergillus sulphureus* KMM 4640 from muddy sand of the eastern Sakhalin shelf (Sea of Okhotsk, 26 m depth) and *Isaria felina* KMM 4639 from sediments (South China Sea, Vietnam shores, 10 m depth), led to the production of five novel prenylated indole alkaloids, 17-hydroxynotoamide D (**1**), 17-O-ethylnotoamide M (**2**), 10-O-acetylsclerotiamide (**3**), 10-O-ethylsclerotiamide (**4**) and 10-O-ethylnotoamide R (**5**) together with known compounds (‒)-notoamide B (**6**), notoamide C (**7**), dehydronotoamide C (**8**), notoamide D (**9**), notoamide F (**10**), notoamide Q (**11**), 17-epi-notoamide Q (**12**), notoamide M (**13**) and sclerotiamide (**14**) (Figure 4) [17]. Among them, compounds **1**–**5** were only produced in the co-culturingprocess.

Compounds **2**, **6**, **8**, **13** and **14** inhibited the proliferation of the human prostate cancer cells 22Rv1 at 100 μM. Notably, **2** and **13** drastically reduced the viability of 22Rv1 prostate cancer cells at 10 μM by 25% and 55%, respectively. 22Rv1 cancer cell lines were resistant to hormone therapy at conventional chemotherapy including two new 2nd generation drugs enzalutamide and abiraterone owing to the presence of the androgen receptor splice variant-7 (AR-V7). Therefore, the active NPs drugs in these cells might be further investigated in the treatment of different human drug-resistant prostate cancer. **6** and **7** displayed weak cytotoxicity against HeLa and L1210 cell lines with half maximal inhibitory concentration (IC_50_) in the range of 22–52 µg/mL [18]. Although **6** and **7** had the similar structure with **9**, compound **9** did not display the similar cytotoxic activity against HeLa and L1210 cell lines. The significant difference in cytotoxicity might be attributed to the possible existence of pyrroloindole system in **9** rather than the dihydroxypyrano-2-oxindole ring system of **6** and **7 [18,19]**. In addition, compounds **1**, **2**, **5**, **9**, **13** and **14** did not exhibit any cytotoxicity against human non-malignant (HEK 293 T and MRC-9) or malignant (PC-3, LNCaP, and 22Rv1) cell lines at concentrations up to 100 μM for 48 h [17].

**Figure 5:**
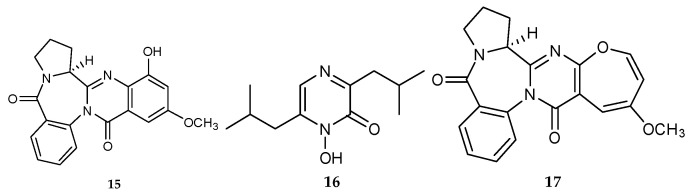
Chemical structures of **15**–**17**.

The co-fermentation of marine mangrove epiphytic fungi *Aspergillus* sp. FSY-01 and FSW-02 collected from a rotten fruit of mangrove *Avicennia marina* in Zhanjiang, Guangdong Province, China, yielded a new alkaloid, aspergicin (**15**), together with two known secondary metabolites, neoaspergillic acid (**16**) and aspergicine (**17**) (Figure 5) [20,21]. Notably, compounds **17** and **15** are chemically isomers, and consequently aspergicine (**17**) may be the precursor of aspergicin (**15**) through a proton 1, 2-shift [22].

Compounds **15** and **16** showed potent inhibitory activities against three Gram-positive bacteria, *Bacillus subtilis* (MIC, minimum inhibitory concentration that inhibits the growth of microbes by 80%, 15.62 and 1.95 μg/mL), *Staphylococcus epidermidis* (MIC 31.25 and 0.49 μg/mL) and *Staphylococcus aureus* (MIC 62.50 and 0.98 μg/mL), and three Gram-negative bacteria, *Escherichia coli* (MIC 31.25 and 15.62 μg/mL), *Bacillus proteus* (MIC 62.50 and 7.80 μg/mL) and *Bacillus dysenteriae* (MIC 15.62 and 7.80 μg/mL), respectively [22].

**Figure 6:**
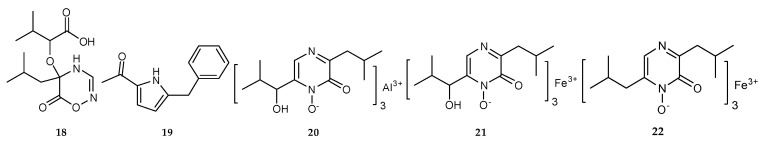
Chemical structures of **18**–**22**.

Marine fungi *Aspergillus sclerotiorum* SCSGAF 0053 and *Penicillium citrinum* SCSGAF 0052 were isolated from the gorgonian corals *Muricella flexuosa* collected from South China Sea, Sanya (18°11′ N, 109°25′ E), Hainan Province, China [23]. Due to the mixed fermentation of marine fungi, a red pigment appeared in the mixed fermentation broth could not be observed in any strain cultured separately. This special phenomenon suggested that a novel biosynthesis route was activated. Four novel alkaloids were obtained, including one oxadiazin derivative sclerotiorumin C (**18**), a pyrrole derivative 1-(4-benzyl-1*H*-pyrrol-3-yl) ethanone (**19**), aluminumneohydroxyaspergillin (**20**) and ferrineohydroxyaspergillin (**21**), together with one known compound ferrineoaspergillin (**22**) (Figure 6) [23]. Compounds **18**–**21** were only produced in the co-culture process.

Compound **20** exhibited potent toxicity towards brine shrimp with medium lethal concentration (LC_50_) value of 6.1 μM and high selective cytotoxicity towards histiocytic lymphoma U937 cell line with an IC_50_ value of 4.2 μM. **19**, **21**, and **22** showed moderate toxicity against brine shrimp with LC_50_ values of 46.2, 11.5 and 27.8 μM, respectively. **21** and **22** possessed mild cytotoxicity against U937 with IC_50_ values of 42.0 and 48.0 μM, respectively. These results suggested that the aluminum complex skeletons of compounds showed more potent toxicity and cytotoxicity than ferricomplex structures of compounds [23,24,25,26]. Moreover, aspergillic acid and **16** also showed more potent inhibitory activities than neohydroxyaspergillic acid and hydroxyaspergillic acid against *B. subtilis*, *E. coli*, *S. aureus* and *Candida albicans* [27].

**Figure 7:**
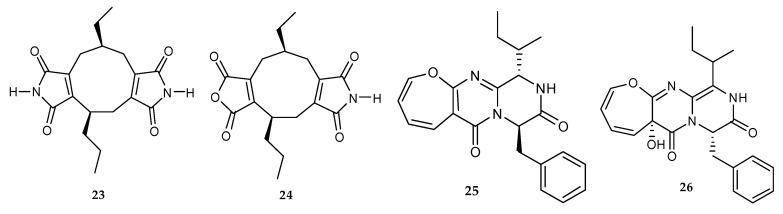
Chemical structures of **23**–**26**.

The co-culture of mangrove fungi *Phomopsis* sp. K38 and *Alternaria* sp. E33 led to the identification of one new diimide derivative, (-)-byssochlamic acid bisdiimide (**23**) and a novel nonadride derivative, (-)-byssochlamic acid imide (**24**) (Figure 7) [28,29]. Ebada et al. (2014) investigated the mycelial extract of a co-cultivation of marine fungal strains *Aspergillus.* BM-05 and BM-05ML, and identified two alkaloids, protuboxepin A (**25**) and oxepinamide E (**26**) (Figure 7) [30]. **23**–**24** were only found in the co-culture process.

Compound **23** exhibited moderate inhibitory activity against HepG2 and Hep-2 with IC_50_ values of 51 μg/mL and 45 μg/mL, respectively. **24** had moderate antifungal activities against *Fusarium oxysporum* and *Fusarium graminearum* with MIC values of 60 μg/mL and 50 μg/mL, respectively [28,29,31]. **25** possessed anti-proliferative activity against human breast cancer adenocarcinoma MDA-MB-231, human acute promyelocytic leukemia HL-60, hepatocellular carcinoma Hep3B, chronic myelogenous leukemia K562 and rat fibroblast 3Y1 cell lines with IC_50_ values of 130, 75, 150, 250 and 180 μM, respectively [32,33]. **26** showed transcriptional activation on liver X receptor α (LXRα) with a half maximal effective concentration (EC_50_) value of 12.8 µM. It was known that LXR was an important target in drug discovery; LXR agonists had been proven to exhibit remarkable therapeutic effects on diabetes, atherosclerosis, Alzheimer’s disease and anti-inflammation. Therefore, **26** was worthy of consideration as a potential lead compound for drug discovery [34].

#### 2.1.2. Alkaloids Derived from the Co-Cultures of Marine Fungi and Bacteria

**Figure 8:**
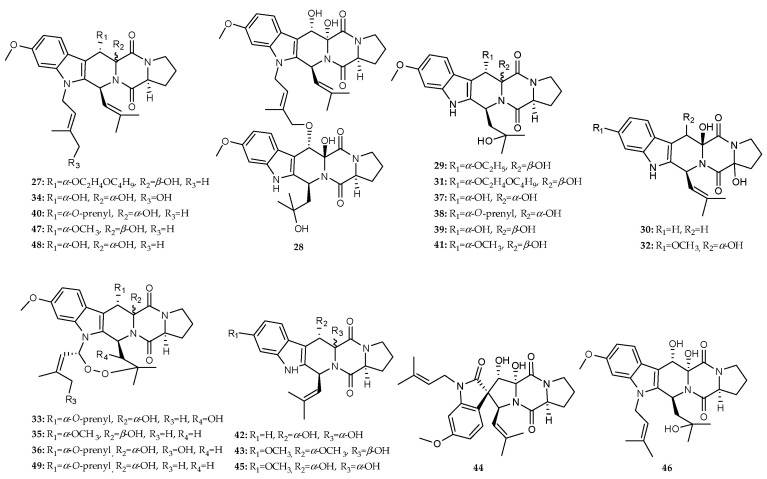
Chemical structures of **27**–**49**.

The alkaloids derived from the co-culture of marine fungi and bacteria were tallied to be 41 isolates (Figure 2D and Figure 3) and can be described as follows; prenylated 2,5-diketopiperazines (2,5-DKPs) were isolated from the co-culture of marine *Penicillium* sp. DT-F29 isolated from marine sediments of Dongtou country, China, and *Bacillus* sp. B31 collected from marine sediments of Changzhi Island, China [35], including ten novel metabolites, 12-*β*-hydroxy-13-butoxyethoxyfumitremorgin B (**27**), diprostatin A (**28**), 12-hydroxy-13*α*-ethoxyverruculogen TR-2 (**29**), hydrocycloprostatin A (**30**), 12-*β*-hydroxy-13*α*-butoxyethoxyverruculogen TR-2 (**31**), hydrocycloprostatin B (**32**), 26-*α*-hydroxyfumitremorgin A (**33**), 25-hydroxyfumitremorgin B (**34**), 12-*β*-hydroxy-13*α*-methoxyverruculogen (**35**), 25-hydroxyfumitremorgin A (**36**) and thirteen known isolates, verruculogen TR-2 (**37**), 12-*α*-hydroxy-13-*α*-prenylverruculogen TR-2 (**38**), 12-hydroxyverruculogen TR-2 (**39**), 13-prenyl fumitremorgin B (**40**), 12-*β*-hydroxy-13-*α*-methoxyverruculogen TR-2 (**41**), cycloprostatin C (**42**), cyclotryprostatin B (**43**), spirotryprostatin C (**44**), 12,13-dihydroxyfumitremorgin C (**45**), neofipiperzine C (**46**), prenylcycloprostatin B (**47**), fumitremorgin B (**48**) and fumitremorgin A (**49**) (Figure 8).

**Figure 9:**
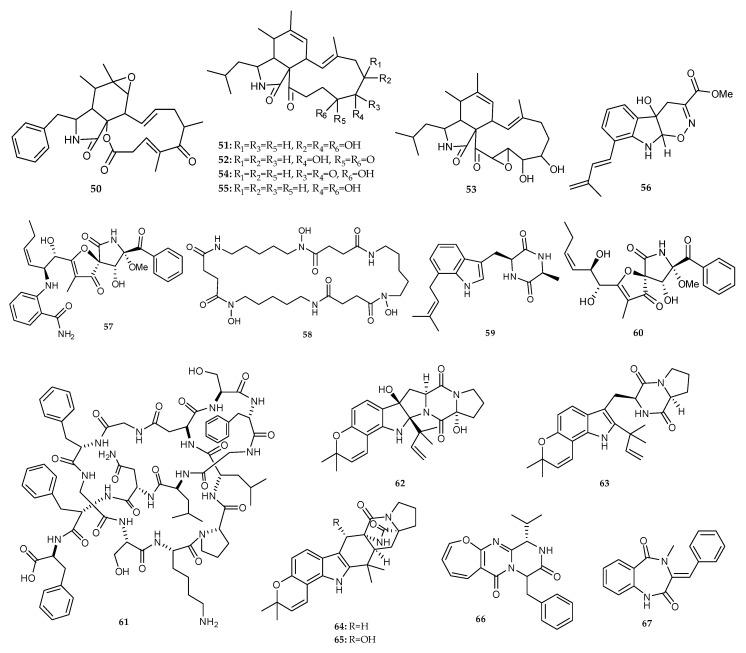
Chemical structures of **50**–**67**.

The secondary metabolites profile of the co-culture of *Streptomyces* sp. and *Aspergillus flavipes*, obtained from marine sediments of the Nanji Islands of the same habitat, showed an induced biosynthesis of a series of known cytochalasans, including rosellichalasin (**50**), and five aspochalasins (aspochalasin E **51**, aspochalasin P **52**, aspochalasin H **53**, aspochalasin M **54** and 19,20-dihydro-aspochalasin D **55**) (Figure 9) [36]. The chromatographic purification of the combination culture extract from marine-derived *Aspergillus fumigatus* MR2012 and *Streptomyces leeuwenhoekii* C34 led to the isolation of two novel compounds, luteoride D (**56**) and pseurotin G (**57**), along with the known isolates, nocardamine (**58**), terezine D (**59**), 11-*O*-methylpseurotin A (**60**) and lasso peptide chaxapeptin (**61**) [37]. In addition, seven known compounds, notoamide D (**9**), speramide B (**62**), notoamide E (**63**), stephacidin A (**64**), notoamide R (**65**), protuboxepin B (**66**) and 3,10-dehydrocyclopeptine (**67**) (Figure 9) were identified from the mixed-fermentation of the marine-derived fungus *Aspergillus versicolor* isolated from sponge *Agelas oroides* and *B. subtilis* [38].

Compounds **27**, **28**, **38**–**40**, **44** and **46**–**49** displayed strong inhibitory effects on bromodomain-containing protein 4 (BRD4) at 20 µM. Notably, **39** and **48** exhibited the most inhibitory activity with 72.7% and 80.4%, compared with the positive control, BRD4 inhibitor (+)-JQ1 (85.7%) [35]. As reported in the previous study, BRD4 protein was a member of the bromodomain and extra-terminal domain (BET) family that carried two bromodomains and was associated with mitotic chromosomes. Bromodomains targeted genetic and epigenetic alterations and regulated chromatin remodeling, which were important therapeutic targets for major diseases, such as neurological disorders, obesity, cancer and inflammation [39,40]. Thus, these compounds further deserved development and research for the treatment of major diseases. Li et al. (2012) reported that **41** had potent inhibitory activities against *Fusarium oxysporum* f. sp. *Niveum*, *Alternaria alternate*, *Fusarium oxysporum* f. sp. *vasinfectum* and *Fusarium solani* with MIC values of 6.25–25 μg/mL and moderate brine shrimp toxicity (LC_50_ 60.7 μg/mL) [41]. The occurrence of **41** could be involved in protecting microbes against invasion by other competing microbes. Therefore, **41** could be considered as a promising lead compound for developing new fungicides. Cui et al. reported **43** could completely inhibit the G2/M phase of tsFT210 cells at concentrations >29.4 µM [42]. Furthermore, Wang et al. (in 2008) showed that **44** had selective cytotoxicity against four cancer cell lines, MOLT-4, HL-60, A-549 and BEL-7402 [43].

Cytochalasans were fungal metabolites that were structurally identified by the presence of a reduced isoindone nucleus connected with a macrocyclic ring [44]. Six cytochalasans (**50**–**55**) showed strong toxicity against *Streptomyces* sp. with 50–80% inhibition at 2–16 μg/mL, and most of them even exhibited 60% inhibition at 2 μg/mL, but they had no any effect on the fungus *A. flavipes* at the same concentration. This indicated that cytochalasans could help *A. flavipes* to compete with *Streptomyces* sp., which was an important support for their potential ecological role. All cytochalasans also exhibited obvious toxicity against human cell lines, as cytochalasans had the ability to inhibit, specifically, the actin filament elongation by blocking the polymerization sites [45,46,47]. Thus, all six compounds (**50**–**55**) exhibited powerful toxicity against *Streptomyces* sp. at 2–16 μg/mL with inhibition rate of 50–80%. Notably, most of these compounds displayed strong inhibitory activity with inhibition rate of 60% even at 2 μg/mL, whereas none of them had antimicrobial activity against the marine-derived producer *A. flavipes* at the same concentration. These findings implied that the co-culture through microbial physical contact could stimulate the expression of silent gene cluster that was responsible for the production of cytochalasans.

The cyclic siderophore, nocardamine (**58**), had inhibitory effects on the proliferation of human tumor cell lines: SK-Mel-5 with an IC_50_ value of 18 μM, T-47D with an IC_50_ value of 6 μM, PRMI-7951 with an IC_50_ value of 14 μM and SK-Mel-28 with an IC_50_ value of 12 μM [48]. Compared with the pure cultures, some novel metabolites were observed in the mixed culture. Two fungal prenylated indole metabolites, **56** and **59**, which were not traced before in *A. fumigatus*, were induced. Both of them had an oxazino [6,5-*b*]indole nucleus which was not previously found in nature. Additionally, the yield of compound **61** was obviously higher than that of the monoculture of *Streptomyces leeuwenhoekii* C58. It was the first time that a bi-lateral cross talk was proved, which resulted in dual induction of both fungal and bacterial metabolites in the same culture conditions. **64** displayed cytotoxic activities toward mouse lymphoma cell line L5178Y with an IC_50_ value of 16.7 μM and in vitro toward testosterone-dependent prostate LNCaP cells with an IC_50_ value of 2.1 μM [49].

#### 2.1.3. Alkaloids Derived from the Co-Cultures of Different Marine Bacteria

**Figure 10:**
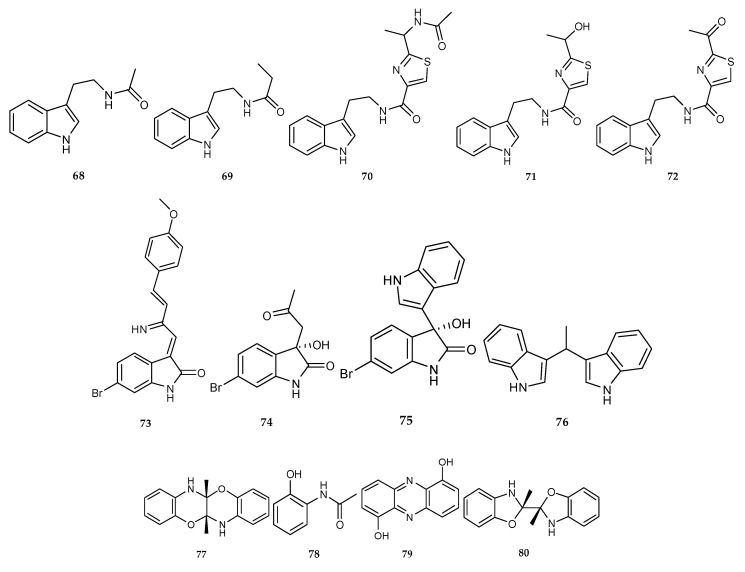
Chemical structures of **68**–**80**.

Thirteen alkaloids were isolated from the co-culture of different marine bacteria (Figure 2E and Figure 3); the structures of these isolates were listed in Figure 10. The average yields of five known tryptamine derivatives, N-acetyltryptamine (**68**), N-propanoyltryptamine (**69**), bacillamide C (**70**), bacillamide B (**71**) and bacillamide A (**72**) using the co-fermentation of marine strain *Streptomyces* sp. CGMCC4.7185 and *Bacillus mycoides* isolated from marine sediments of the Nanji Island (China, 27°42′ N, 121°08′ E), were 14.9, 2.8, 9.6, 13.7 and 3.0 mg/L, respectively, which were all undetectable under simple culture conditions [50]. This was the first report of applying a microorganism co-culture system to enhance the yields of known compounds [50].

In 2018, El-Hawary et al. identified four indole alkaloids—a novel brominated oxindole alkaloid saccharomonosporine A (**73**), a novel convolutamydine F (**74**) and two known compounds, (*S*) 6-bromo-3-hydroxy-3-(1H-indol-3-yl) indolin-2-one (**75**) and vibrindole (**76**)—from the mixed fermentation culture of two sponge-associated actinomycetes, *Saccharomonospora* sp. UR22 and *Dietzia* sp. UR66 collected from the Red Sea sponge *Callyspongia siphonella* [51].

Two sponge-associated actinomycetes, *Actinokineospora* sp. EG49 isolated from the Red Sea sponge, *Spheciospongia vagabunda*, and *Nocardiopsis* sp. RV163 derived from the Mediterranean sponge, *Dysidea avara*, were co-cultivated together and yielded a novel 5a,6,11a,12-tetrahydro-5a,11a-dimethyl-1,4-benzoxazino[3,2-*b*][1,4]benzoxazine (**77**) and three known metabolites, N-(2-hydroxyphenyl)-acetamide (**78**), 1,6-dihydroxyphenazine (**79**) and 2,2′,3,3′-tetrahydro-2,2′-dimethyl-2,2′-bibenzoxazole (**80**) [52].

Pim-1 kinase is a well-established oncoprotein in several tumor entities, such as prostate cancer, pancreatic cancer, colorectal cancer and myeloid leukemia. Inhibition of Pim-1 kinase would prevent the growth of tumor cells. Compounds **73** and **75** exhibited potent Pim-1 kinase inhibitors with IC_50_ values of 0.3 μΜ and 0.946 μΜ, respectively. Docking studies showed the binding model of **73** and **75** in the ATP pocket of Pim-1 kinase. They also exhibited obvious antiproliferative activity against human promyelocytic leukemia HL-60 (IC_50_ 2.8 and 4.9 µΜ) and human colon adenocarcinoma HT-29 (IC_50_ 3.6 and 3.7 μΜ). This indicated that **73** and **75** could act as potential Pim-1 kinase inhibitors that mediated the inhibitory effects on the growth of tumor cells [51].

In addition, only compound **79** was documented against *Trypanosoma brucei* (IC_50_ 19 μΜ), *Bacillus* sp. (11 mm inhibition zone diameter) and *Actinokineospora* sp. EG49 (15 mm inhibition zone diameter) [52]. The yield of **79** was very high in the co-culture process. However, it was not detected in the single microbial culture. Co-culture strategy not only enhanced the chemical diversity of the metabolites but also increased the production of metabolites undetected in the single microbial culture.

### 2.2. Anthraquinones

**Figure 11:**
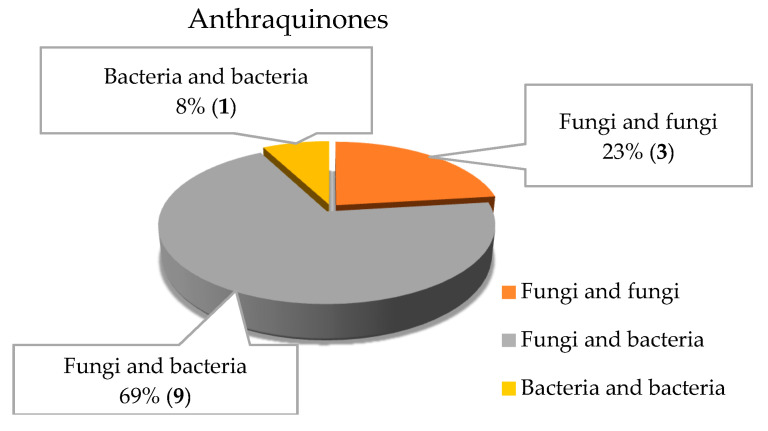
Anthraquinones isolated from the co-cultures of marine fungi–fungi, fungi–bacteria and bacteria–bacteria.

Thirteen different anthraquinone isolates were obtained from different marine microbial co-cultures; the co-cultures of marine fungi–bacteria represented the majority, 69% (9/13 isolates; Figure 2B and Figure 11).

#### 2.2.1. Anthraquinones Derived from the Co-Cultures of Different Marine Fungi

**Figure 12:**
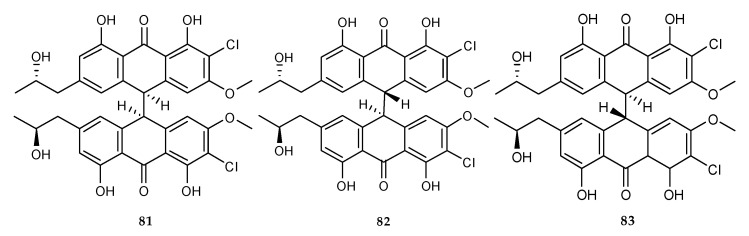
Chemical structures of **81**–**83**.

In the recent study, the combination of cultures from two different developmental stages of marine alga-derived *Aspergillus alliaceus* (teleomorph: *Petromyces alliaceus*) drastically changed the metabolite profile and resulted in the production of allianthrone A (**81**) and two diastereomers, allianthrones B (**82**) and C (**83**) (Figure 12) [53]. **81**–**83** exhibited cytotoxic activity against SK-Mel-5 melanoma cell lines with IC_50_ (11.0, 12.2, and 19.7 μM) and HCT-116 colon carcinoma cells with IC_50_ (9.0, 10.5 and 13.7 μM), respectively. This study presented the first example of elicitation of novel fungal chemical diversity by a co-existing strategy of two different developmental phenotypes of *Aspergillus* species. For several Aspergilli, e.g., *A. alliaceus*, asexual and sexual life developmental stages were known. However, rarely did they co-cultivate at the same time. Even more surprising was the presence of novel bianthrones when the sclerotial and asexual morphs of the same species co-existed. There were only a few examples that showed differences in secondary metabolites in fungi based on their distinct developmental stages or chemical profiles for the two mating types of heterothallic fungi. However, none of these compounds displayed any activity against *P. aeruginosa*, *E. faecium*, *S. aureus*, *E. coli*, *C. albicans* and *B. subtilis.* Furthermore, non-significant results were obtained against lung (A549), prostate (PC3) and breast (MCF-7) human cancer cells compared with the positive control, etoposide [53].

#### 2.2.2. Anthraquinones Derived from the Co-Cultures of Marine Fungi and Bacteria

**Figure 13:**
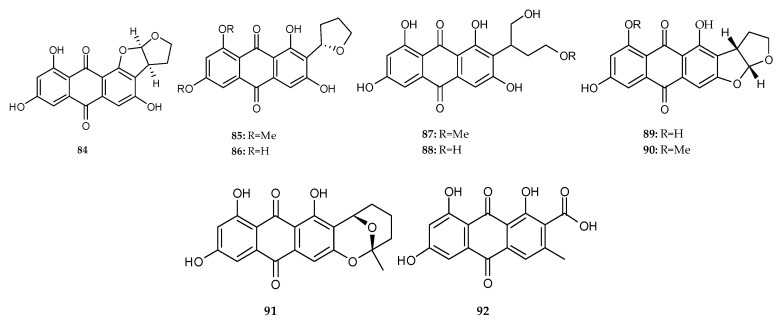
Chemical structures of **84**–**92**.

Two novel anthraquinones, (*z*)(11*S*,12*R*)-versicolorin B (**84**) and 6,8-O-dimethylbipolarin (**85**), along with seven known substances bipolarin (**86**), versiconol (**87**), versiconol acetate (**88**), versicolorin B (**89**), 8-*O*-Methylversicolorin B (**90**), averufin (**91**) and endocrocin (**92**) (Figure 13) were isolated and identified from the mixed fermentation broth of the marine fungus *A. versicolor* and *B. subtilis* [38].

Versiconol (**87**) was characterized as an inhibitor of protein tyrosine kinases against EGF-R and v-abl protein tyrosine kinases that were responsible for catalyzing phosphorylation of tyrosine residues of protein substrates, and suppression of MK-cells [54]. **89** displayed inhibitory activity against the Gram-positive *S. aureus* with MIC value of 50 μM and antifungal activity against *Fusarium solani* with MIC values of 16–32 µg/mL [38,55]. The cytotoxic bioassay of **90** was recorded against mouse lymphoma cell line L5178Y with an IC_50_ value of 21.2 μM. Moreover, **91** displayed antibacterial activity against *B. subtilis* (MIC = 8–16 μg/mL) and the Gram-positive *S. aureus* (MIC = 25 μM) and four Gram-positive microbes, including two *E. faecalis* and two *E. faecium* (MIC = 12.5–25 μM) [38,55]. Neither **89** nor **91** had cytotoxicity against L5178Y cell line, which implied that their antimicrobial activities were not associated with their respective general toxicities. Besides, **90** also displayed mild cytotoxic activity against human lung cancer cells H460 and the human prostate cancer cells PC-3 with IC_50_ values of 27.2 and 19.5 μM, respectively [56]. Other compounds did not exhibit distinct cytotoxic activity against L5178Y cell line and antibacterial activity against five Gram-positive microbes, including one *S. aureus*, two *E. faecalis* and two *E. faecium*.

#### 2.2.3. Anthraquinones Derived from the Co-Cultures of Different Marine Bacteria

**Figure 14:**
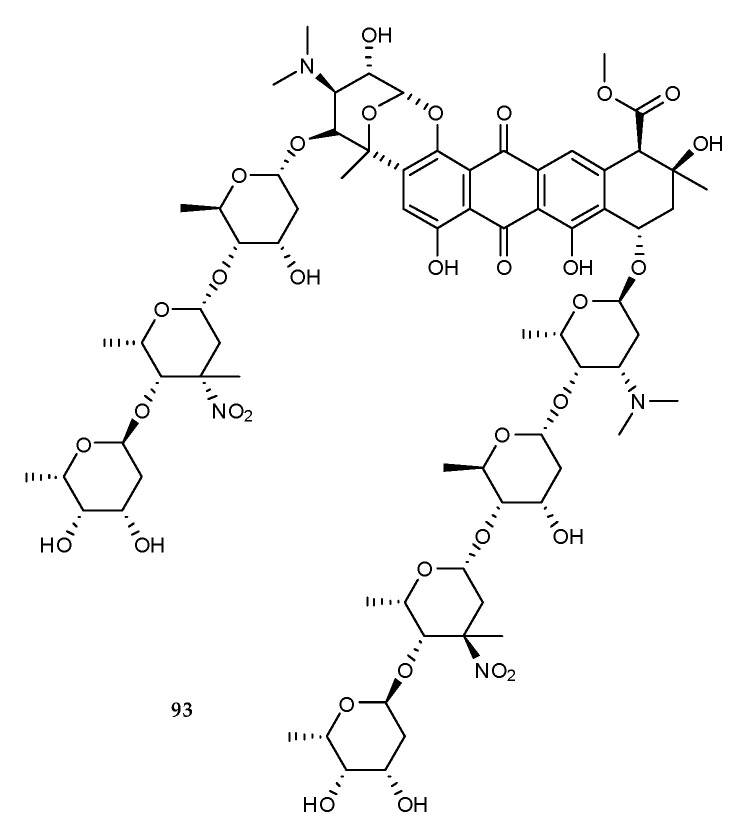
Chemical structures of **93**.

A new antibiotic, keyicin (**93**) (Figure 14), was purified and identified from a co-culture of two marine invertebrate-associated bacteria *Micromonospora* sp. WMMB-235 and *Rhodococcus* sp. WMMA-185 [57]. It showed selective inhibitory activity against Gram-positive bacteria and could inhibit the growth of *B. subtilis* and Methicillin Sensitive *Staphylococcus aureus* (MSSA) with MIC values of 9.9 μM and 2.5 μM, respectively. In contrast to many anthracyclines, **93** might modulate fatty acid metabolism and exhibit antibacterial activity without nucleic acid damage that is explained by keyicin’s mechanism of action (MOA) based on *E. coli* chemical genomics studies [57].

### 2.3. Cyclopeptides

Cyclopeptides are cyclic compounds mainly formed by the amide bonds of proteinogenic or non-proteinogenic amino acids bound together. Several fungal cyclic peptides have been developed as pharmaceuticals, such as the echinocandins, pneumocandins and cyclosporin A [58]. Six cyclopeptides were produced by the co-cultures of marine fungi–fungi (four isolates, 67%) and fungi–bacteria (two isolates, 33%) from different marine sources. However, marine bacteria–bacteria did not yield these structures in this period of investigation.

#### 2.3.1. Cyclopeptides Derived from the Co-Cultures of Different Marine Fungi

**Figure 15:**
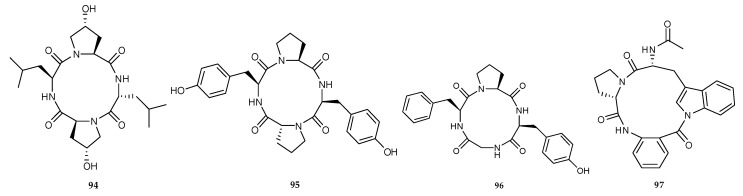
Chemical structures of **94**–**97**.

Three new cyclic tetrapeptides, named cyclo-(L-leucyl-*trans*-4-hydroxy-L-prolyl-D-leucyl-*trans*-4-hydroxy-L-proline) (**94**) [59] cyclo (D-Pro-L-Tyr-L-Pro-L-Tyr) (**95**) and cyclo (Gly-L-Phe-L-Pro-L-Tyr) (**96**) (Figure 15) [60] were identified from the co-culture of two mangrove fungi *Phomopsis* sp. K38 and *Alternaria* sp. E33 isolated from the South China Sea. Meanwhile, the co-cultivation of two marine alga-derived fungi *Aspergillus* sp. BM-05 and BM-05ML isolated from a brown algal species collected off Helgoland, North Sea, Germany, yielded a new cyclotripeptide, psychrophilin E (**97**) (Figure 15) [30].

Compound **94** exhibited in vitro moderate to high inhibitory activity towards four crop-threatening fungi, *Helminthosporium sativum*, *Gaeumannomyces graminis*, *F. graminearum* and *Rhizoctonia cereals* with MIC values of 130, 220, 250 and 160 μg/mL, respectively [59]. **95** and **96** showed high in vitro antifungal activity against human fungus (*Candida albicans*) with MIC values of 35 μg/mL and 25 μg/mL, respectively [60]. **97** exhibited anti-proliferative activities against four human cancer cells, human cisplatin-resistant ovarian cancer A2780CisR, colon carcinoma HCT116, ovarian cancer A2780 and chronic myelogenous leukemia K562 with IC_50_ values of 49.4, 28.5, 27.3 and 67.8 µM, respectively. The inhibition of HCT116 cells by **97** was more potent than that of the positive control, cisplatin (IC_50_ 33.4 µM) [30].

#### 2.3.2. Cyclopeptides Derived from the Co-Cultures of Marine Fungi and Bacteria

**Figure 16:**
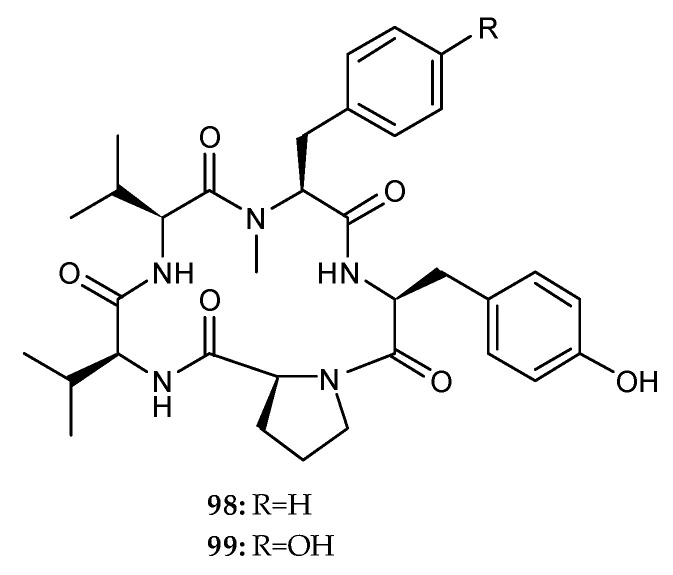
Chemical structures of **98**–**99**.

Recently, the chemical investigation of the mixed-fermentation of a marine fungus *Aspergillus versicolor* isolated from the sponge *Agelas oroides* and *B. subtilis* yielded two cyclic pentapeptides, one new cotteslosin C (**98**) and a known cotteslosin A (**99**) (Figure 16) [38]. Both of them did not show significant cytotoxic activity towards mouse lymphoma cell line L5178Y, or even antibacterial activity against five Gram-positive microbes, including one *S. aureus*, two *E. faecalis* and two *E. faecium* [38]. **99** displayed weak cytotoxicity against another three human cancer cell lines, prostate DU145, melanoma MM418c5 and breast T47D, with EC_50_ values of 90, 66 and 94 µg/mL, respectively [61].

### 2.4. Macrolide

There were no reported macrolides from the co-cultures of marine fungi–fungi and fungi–bacteria. Only one isolate was identified from a co-culture of marine bacteria–bacteria.

#### Macrolides Derived from the Co-Cultures of Different Marine Bacteria

**Figure 17:**
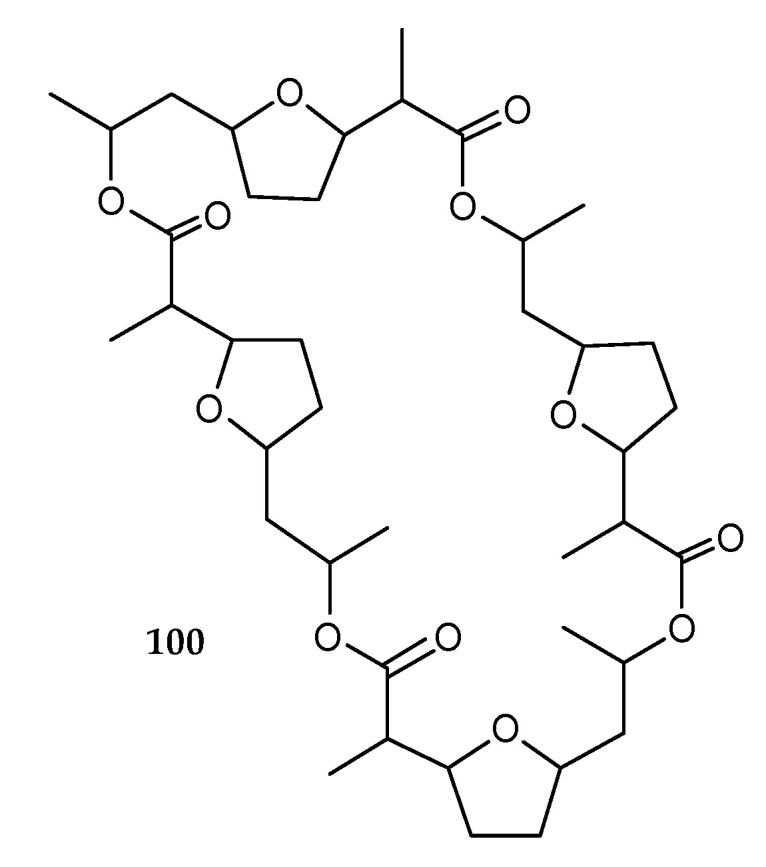
Chemical structures of **100**.

A known compound, nonactin (**100**) (Figure 17) was isolated from the co-culture of two marine bacteria, *Saccharomonospora* sp. UR22 and *Dietzia* sp. UR66 [51]. It possessed a macrotetrolide structure integrated from nonactic acid, and exhibited antitumor and antibacterial activity, especially its inhibitory effects on the P170 glycoprotein-mediated efflux of chemotherapeutic agents in multiple-drug-resistant cancer cells [62,63,64,65].

### 2.5. Phenylpropanoids

Phenylpropanoids are a big and structurally diverse group of secondary metabolites, which bear a C_6_–C_3_ phenolic scaffold that play crucial roles in a wide spectrum of biological and pharmacological activities [66]. Twenty-three phenylpropanoids were isolated from co-culture of marine fungi–fungi (12 isolates, 52%) and fungi–bacteria (11 isolates, 48%), while there are no reported phenylpropanoids from the co-culture of different marine bacteria.

#### 2.5.1. Phenylpropanoids Derived from the Co-Cultures of Different Marine Fungi

**Figure 18:**
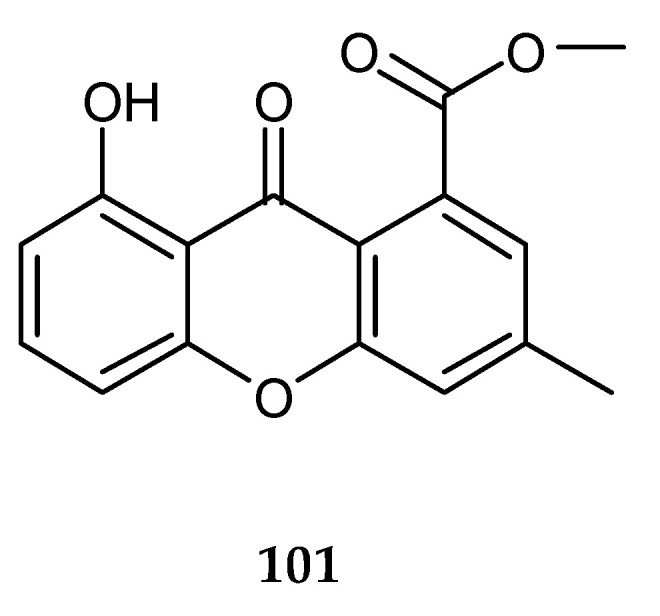
Chemical structures of **101**.

A xanthone derivative known as 8-hydroxy-3-methyl-9-oxo-9*H*-xanthene-1-carboxylic acid methyl ether (**101**) (Figure 18) was discovered from the mixed culture of two mangrove fungi, *Phomopsis* sp. K38 and *Alternaria* sp. E33 [67] from the South China Sea coast. It showed a broad spectrum of antifungal activities against plant pathogens, *Blumeria graminearum*, *Gloeasporium musae*, *F. oxysporum*, *Colletotrichum glocosporioides* and *Peronophthora cichoralearum*.

**Figure 19:**
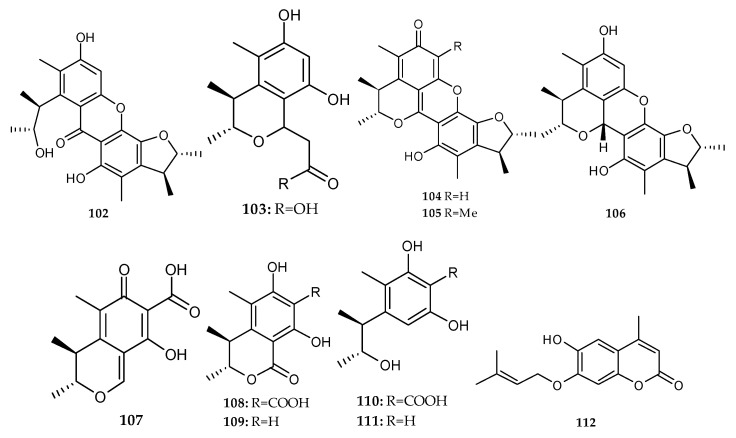
Chemical structures of **102**–**112**.

Ten citrinin analogues were isolated and identified from the co-culture of two marine algal-derived endophytic fungal strains, *Aspergillus sydowii* EN-534 and *Penicillium citrinum* EN-535 collected from marine red alga *Laurencia okamurai*, including two novel compounds, citrinin dimer *seco*-penicitrinol A (**102**) and citrinin monomer penicitrinol L(**103**), and the known penicitrinone A (**104**), penicitrinone F (**105**), penicitrinol A (**106**), citrinin (**107**), dihydrocitrinone (**108**), decarboxydihydrocitrinone (**109**) phenol A acid (**110**) and phenol A (**111**) (Figure 19) [68]. In addition, one novel coumarin named 7-(γ,γ-dimethylallyloxy)-6-hydroxy-4-methylcoumarin (**112**) (Figure 19) was detected and characterized from the co-culture of the two mangrove fungi, *Phomopsis* sp. K38 and *Alternaria* sp. E33 [69].

Compounds **104**, **106** and **107** exhibited inhibitory activities against two human pathogens *Micrococcus luteus* and *E. coli*, and three aquatic bacteria *Vibrio parahaemolyticus*, *Vibrio alginolyticus* and *Edwardsiella ictaluri* with MIC values of 4–64 μg/mL. **102**, **103** and **105** inhibited *V. alginolyticus* and *E. ictaluri* with MIC values of 32–64 μg/mL. **103** and **105** inhibited *V. parahaemolyticus* and *E. coli* with MIC values of 32 and 64 μg/mL, respectively. Moreover, **102**–**107** were further evaluated for anti-influenza neuraminidase (homologous protein of H_5_N_1_) activity. **104** and **105** exhibited significant inhibitory activities with IC_50_ values of 12.9 and 18.5 nM, respectively [68]. Thus, these bioactive substances could be further optimized for the development of antibacterial and anti-influenza agents. In addition to the anti-influenza activity, the activated metabolite penicitrinone A (**104**) also exerted an inhibitory effect on four human cancer cell lines, HL-60, K562, BGC-823 and HeLa cells with IC_50_ values of 43.2, 50.8, 54.2 and 65.6 μM, respectively [70].

#### 2.5.2. Phenylpropanoids Derived from the Co-Cultures of Marine Fungi and Bacteria

**Figure 20:**
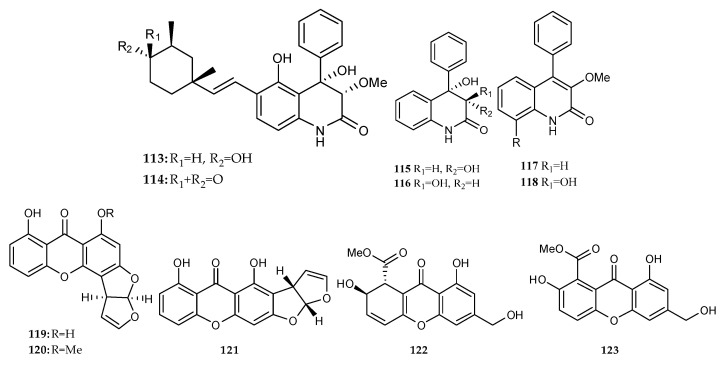
Chemical structures of **113**–**123**.

The chemical investigation of the mixed culture of the marine fungus *A. versicolor* and *B. subtilis* resulted in the isolation of one novel aflaquinolone, 22-epi-aflaquinolone B (**113**); and ten known metabolites, aflaquinolone A, F and G (**114**–**116**), 3-*O*-methylviridicatin (**117**), 9-hydroxy-3-methoxyviridicatin (**118**), *O*-demethylsterigmatocystin (**119**), sterigmatocystin (**120**), sterigmatin (**121**), AGI-B4 (**122**) and sydowinin B (**123**) (Figure 20) [38].

The metabolite 3-*O*-methylviridicatin (**117**) was reported to possess inhibitory activity against human immunodeficiency virus (HIV) (Heguy et al., 1998). It could prevent cytokine tumor necrosis factor α (TNF-α), induce the HIV expression with long terminal repeat in HeLa cells (IC_50_, 5μM) and block the viral replication in the model of chronic infection in OM-10.1 cell lines which directed at the induction of TNF-α [71]. **119** exhibited cytotoxic activities towards mouse lymphoma cell line L5178Y with an IC_50_ value of 5.8 μM. Three xanthone derivatives (**120**–**122**) showed potent cytotoxic activities towards the mouse lymphoma cell lines with IC_50_ values of 2.3, 2.2 and 2.0 μM, respectively, compared with a positive control, kahalalide F (IC_50_ = 4.3 μM). Sterigmatocystin (**120**) also exhibited strong cytotoxicity towards human hepatoma cells (HepG2) at 3 μM [72]. Its mechanism suggested that it could stimulate a biotransformation process, increase the population of reactive oxygen species and promote the imbalance in the antioxidant defense system caused by the process of lipid peroxidation [73]. Recently, Zingales et al. (2020) displayed the significant role of mitochondria in sterigmatocystin-induced toxicity in SH-SY5Y cells [74]. The reduced viability of SH-SY5Y cells displayed time- and dose-dependence with mitochondrial dysfunction when exposed to **120** in response to the forced dependency of the cells on mitochondrial oxidative phosphorylation [74]. Thus, these findings provided us a valuable direction for the application of neuroprotective mitochondria-target functional peptides. Moreover, compound **122** inhibited human umbilical vein endothelial cells (VEGF-induced proliferation of HUVECs) with an IC_50_ value of 1.4 μM [75]. It is considered as a novel inhibitor of vascular endothelial cell growth factor, which is one of the main stimulants of angiogenesis.

### 2.6. Polyketides

**Figure 21:**
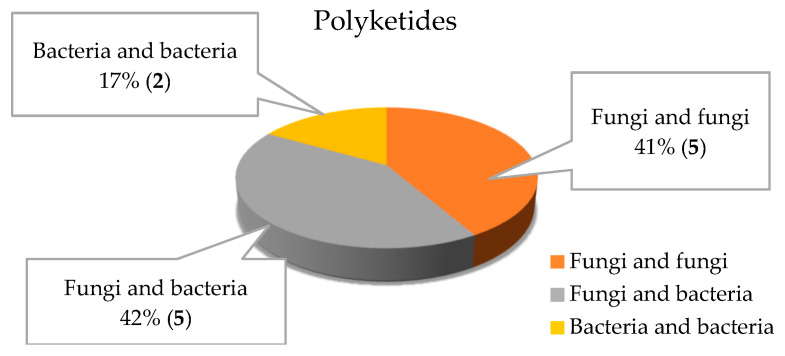
Polyketides isolated from the co-culture of marine fungi–fungi, fungi–bacteria and bacteria–bacteria.

Twelve polyketides were isolated and characterized from the marine microbial co-cultures in recent years (Figure 2B and Figure 21).

#### 2.6.1. Polyketides Derived from the Co-Cultures of Different Marine Fungi

**Figure 22:**
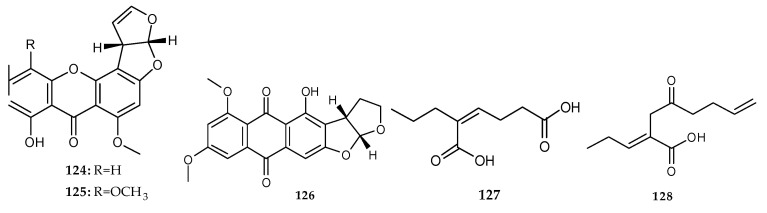
Chemical structures of **124**–**128**.

In 2014, Ebada et al. identified three previously reported polyketide derivatives, sterigmatocystin (**124**), 5-methoxysterigmatocystin (**125**) and aversin (**126**) (Figure 22) from the ethyl acetate extract of two marine alga-derived fungi, *Aspergillus* sp. BM-05 and BM-05ML [30]. Kossuga et al. isolated two new and unusual polyketides: (*Z*)-2-ethylhex-2-enedioic acid (**127**) and (*E*)-4-oxo-2-propylideneoct-7-enoic acid (**128**) (Figure 22) from the marine-derived fungi *Penicillium* sp. Ma(M3)V isolated from the marine sponge *Mycale angulosa* co-cultivated with *Trichoderma* sp. Gc(M2)1 isolated from the marine sponge *Geodia corticostylifera* [76]. Two unprecedented polyketides (**127**–**128**) had a common feature—a conjugated carboxylic acid group that could be biogenetically generated from the methyl group of an acetate rather than a methionine precursor in **127**, and the same group could be derived from C-1 position of an acetate or C-2 position of a propionate in **128** based on the precursor of the ethyl group connected to a double bond. It was an excellent case of a truly novel carbon skeleton induced by the powerful and underexplored method, marine microbial co-cultivation.

Compound **124** showed in vitro anti-proliferative activities towards human cisplatin-resistant ovarian cancer A2780CisR, ovarian cancer A2780 and chronic myelogenous leukemia K562 cell lines with IC_50_ values of 95.5, 30.6 and 57.0 µM, respectively. Moreover, it also displayed more significant anti-proliferative activities against human colon carcinoma HCT116 cells with an IC_50_ value of 10.3 µM (cf. to cisplatin’s IC_50_ 33.4 µM). Compound **125** exhibited potent in vitro anti-proliferative activities towards three human cancer cell lines, HCT116, A2780 and human chronic myelogenous leukemia (K562) with IC_50_ values of 4.4, 51.0 and 13.4 µM, respectively [30].

#### 2.6.2. Polyketides Derived from the Co-Cultures of Marine Fungi and Bacteria

**Figure 23:**
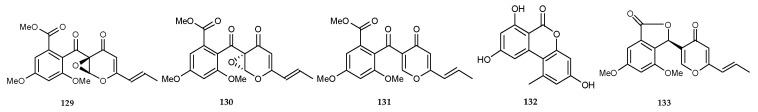
Chemical structures of **129**–**133**.

A pair of enantiomers (9*R*,14*S*)-epoxy-11-deoxyfunicone (**129**) and (9*S*,14*R*)-epoxy-11-deoxyfunicone (**130**), along with deoxyfunicone (**131**), alternariol (**132**) and vermistatin (**133**) (Figure 23) were isolated from the co-culture of *Penicillium* sp. WC-29-5 isolated from the mangrove soil around the roots of *Aegiceras corniculatum* and *Streptomyces fradiae* 007 isolated from a sediment sample in the Jiaozhou Bay, Shandong Province, China [77].

Both **129** and **130** exhibited moderate inhibitory activity against H1975 tumor cell lines with IC_50_ values of 3.97 and 5.73 μM, respectively. Deoxyfunicone (**131**) was found to exert anti-inflammatory activity, exhibiting the inhibition effect on overproduction of nitric oxide (NO) and the prostaglandin E_2_ in both lipopolysaccharide-provoked BV2 microglial and lipopolysaccharide-stimulated RAW264.7 macrophage cells (IC_50_ = 10.6 and 40.1 μM, respectively) [78]. **132** was known as a cytotoxic, genotoxic, mutagenic and fetotoxic mycotoxin [79,80]. However, in the IL-1β-stimulated Caco-2 cells, the metabolite **132** increased the transcription of TNF-α; inversely reduced the transcription of IL-1β and IL-6; and decreased the transcription and secretion of IL-8, suggesting that **132** possessed immunomodulatory activities on both lipopolysaccharide- and IL-1 β-related pathways in non-immune intestinal epithelial cells [79].

#### 2.6.3. Polyketides Derived from the Co-Cultures of Different Marine Bacteria

**Figure 24:**
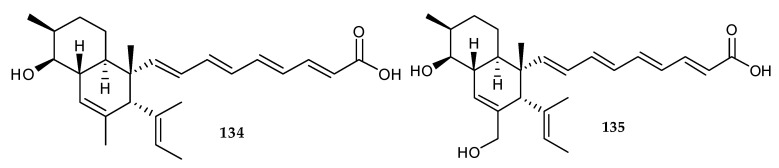
Chemical structures of **134**–**135**.

Recently, two unusual polyketides, janthinopolyenemycins A (**134**) and B (**135**) (Figure 24) were purified and identified from the co-cultivation broth of two marine bacteria *Janthino bacterium* spp. ZZ145 and ZZ148 isolated from marine soil sample [81]. Both **134** and **135** displayed the same antifungal activity against *C. albicans* with a minimum bactericidal concentration (MBC) value of 31.25 μg/mL and an MIC value of 15.6 μg/mL. However, none of them could suppress the growth of methicillin-resistant *S. aureus* or *E. coli* (MIC > 100 μg/mL) [81].

### 2.7. Steroids

Steroids contain a characteristic arrangement of four cycloalkane rings that are joined together. They represent a large family of compounds that play important roles as chemical messengers, and the scaffold is present in many FDA-approved drugs [82,83,84]. A total of five steroidal metabolites were reported; four of them were isolated from the co-culture of marine fungi–bacteria (80%); only one isolate was identified from the co-culture of marine fungi–fungi (20%). No isolates were obtained from the co-culture of marine bacteria–bacteria.

#### 2.7.1. Steroids Derived from the Co-Cultures of Different Marine Fungi

**Figure 25:**
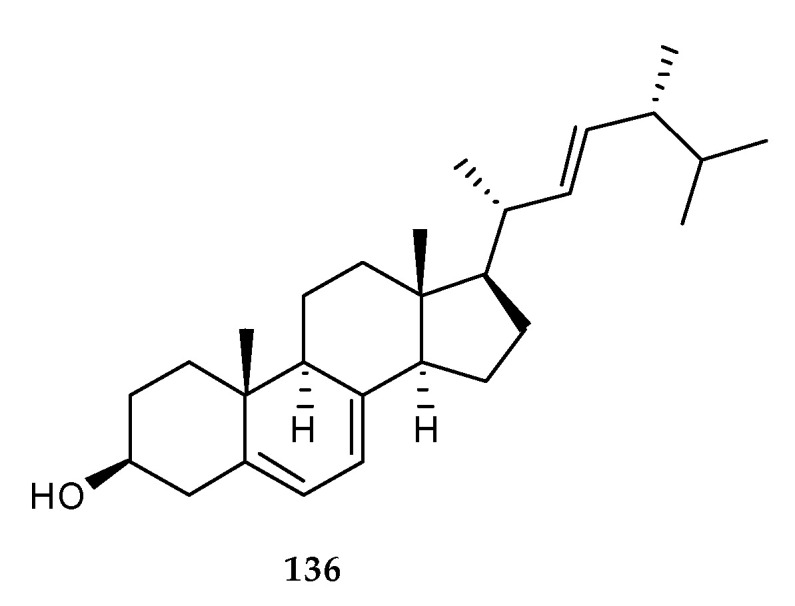
Chemical structures of **136**.

To the best of our knowledge, the only one steroid, ergosterol (**136**), was found from the co-culture broth of two marine mangrove epiphytic fungi, *Aspergillus* sp. FSY-01 and FSW-02 (Figure 25) [21,85]. It was an essential component of fungal cell membrane with strong specificity and stable structure. Therefore, **136** was widely applied to detecting fungal containment as an indicator of fungal biomass [86].

#### 2.7.2. Steroids Derived from the Co-Cultures of Marine Fungi and Bacteria

**Figure 26:**
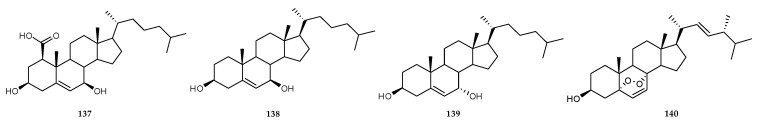
Chemical structures of **137**–**140**.

An unprecedented steroid, 7*β*-hydroxycholesterol-1*β*-carboxylic acid (**137**), together with three known steroidal metabolites, 7*β*-hydroxycholesterol (**138**), 7*α*-hydroxycholesterol (**139**) and ergosterol-5*α*,8*α*-peroxide (**140**) (Figure 26), have been confirmed from the co-culture of two marine alga-derived microbes, *Aspergillus* sp. BM05, and an unidentified bacterium (BM05BL), isolated from the brown alga of the genus *Sargassum* collected off Helgoland, North Sea, Germany [87].

Compounds **137**–**140** showed moderate activities against four human tumor cell lines, A2780, HCT116, K562 and A2780 CisR with the IC_50_ values of 10.0–100.0 µM. At the same time, the total extract of co-culture of *Aspergillus* sp. BM05 and BM05BL showed obvious antiproliferative activity compared with its single steroidal compounds. This implied a synergistic role of these steroidal metabolites in the extract. Furthermore, **140** was reported as a promising new candidate that could overcome the drug-resistant property of malignant cancer cells through abolishing miR-378, a microRNA involved in new tumor initiation, unlimited self-renewal and recurrence of tumor cells after chemotherapy [88].

### 2.8. Terpenoids

Terpenoids known as isoprenoids are structurally diverse metabolites found in many natural sources. This class of compounds displays a wide sector of important pharmacological entities that confirmed by several preclinical and clinical studies [89,90]. Only two terpenoidals were isolated from the co-cultures of marine fungi–bacteria (one compound, 50%) and bacteria–bacteria (one compound, 50%).

#### 2.8.1. Terpenoids Derived from the Co-Cultures of Marine Fungi and Bacteria

**Figure 27:**
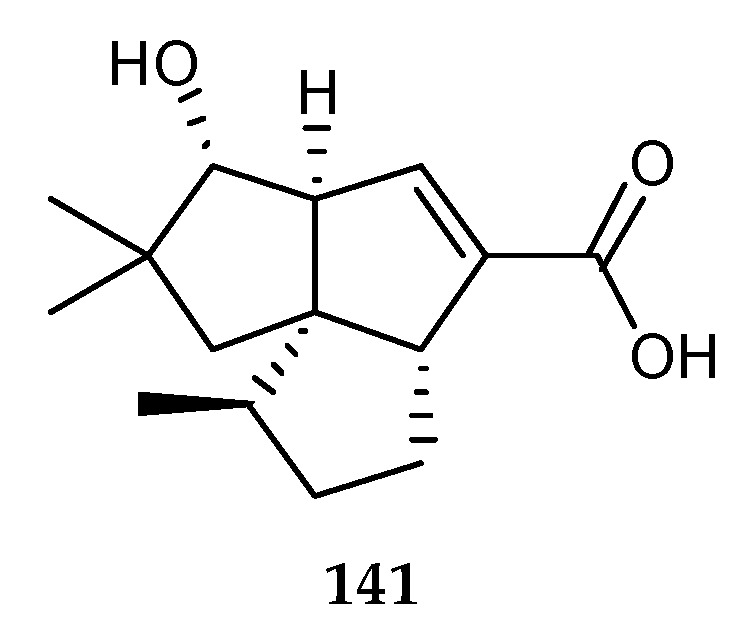
Chemical structures of **141**.

The production of the bacterial sesquiterpene pentalenic acid (**141**) (Figure 27) might be attributed to the competition relationship between marine fungus *A. fumigatus* MR2012 isolated from a Red Sea sediment in Hurghada, Egypt and terrestrial bacterium *S. leeuwenhoekii* C58 collected from the hyper-arid soil of Laguna de Chaxa Salar de Atacama, Chile, in which *S. leeuwenhoekii* C58 suppressed the production of *A. fumigatus* MR2012 and enhanced the production of **141 [37]**. This suggested that *S. leeuwenhoekii* C58 appeared to activate the cryptic biosynthetic gene clusters to construct a defense mechanism based on the chemical signals generated by the competitive fungus, *A. fumigatus* MR2012. Thus, the bacterial strain was capable of suppressing the biosynthesis of the fungus metabolites that were present in the axenic cultures.

#### 2.8.2. Terpenoids Derived from the Co-Cultures of Different Marine Bacteria

**Figure 28:**
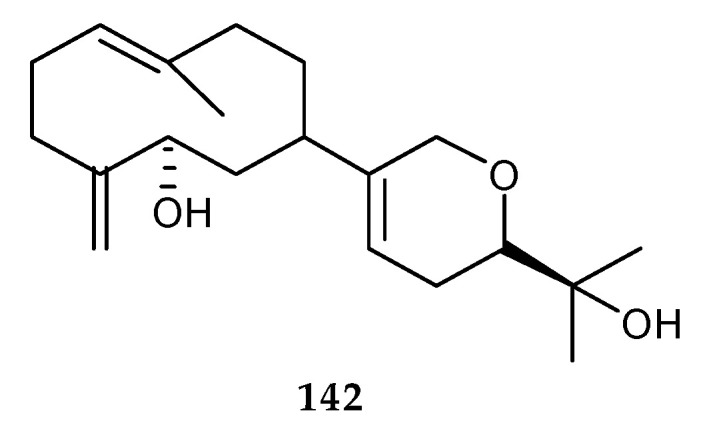
Chemical structures of **142**.

A diterpene lobocompactol (**142**) (Figure 28) was isolated from the co-culture of marine actinomycete *Streptomyces cinnabarinus* PK209 collected from the seaweed rhizosphere, obtained at a depth of 10 m along the coast of Korea and its competitor *Alteromonas* sp. KNS-16. Its productivity was increased 10.4-fold higher than that of the pure culture of PK209 [91]. Moreover, its antifouling activities were recently confirmed against primary fouling organisms, including diatoms, bacteria, and macroalgae zoospores. In order to further determine whether **142** was a non-toxic antifoulant, the therapeutic rate (LC_50_/EC_50_) was used to evaluate its toxicity, the LC_50_/EC_50_ of **142** was more than that of **15**, indicating that the metabolite **142** was a non-toxic antifoulant. Thus, this compound could be valuable as an antifouling agent in both antifouling coating industry and marine ecology.

### 2.9. Others

Twelve compounds with other structures were obtained by co-culture of marine fungi–bacteria (4 compounds, 33%) and fungi–fungi (8 compounds, 67%).

#### 2.9.1. Other Compounds Derived from the Co-Cultures of Different Marine Fungi

**Figure 29:**
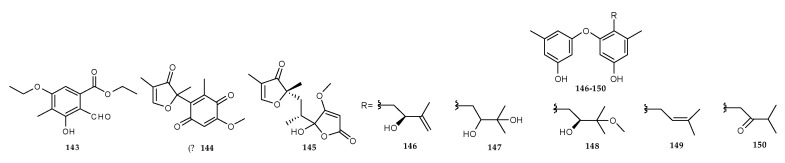
Chemical structures of **143**–**150**.

A novel polysubstituted benzaldehyde derivative, ethyl-5-ethoxy-2-formyl-3-hydroxy-4- methylbenzoate (**143**) (Figure 29) was identified from the mixed fermentation of the two mangrove fungi, *Phomopsis* sp. K38 and *Alternaria* sp. E33 that were collected from the South China Sea [92]. Another two novel furanone derivatives were identified as sclerotiorumins A and B (**144**, **145**) (Figure 29) from the co-culture of the two marine fungi, *sclerotiorum* SCSGAF 0053 and *P. citrinum* SCSGAF 0052 isolated from gorgonian *Muricella flexuosa* collected from the South China Sea, Sanya (18°11′ N, 109°25′ E), Hainan Province, China [23]. Five diorcinols, including one novel diorcinol J (**146**) and four known diorcinols B-E (**147**–**150**) (Figure 29), were characterized during the co-culturing of two marine-derived fungi, *A. sulphureus* KMM 4640 and *I. felina* KMM 4639 [93].

Compound **143** showed in vitro inhibitory activity against *G. musae*, *F. graminearum*, *P. sojae* (*Kaufmann* and *Gerdemann*) and *Rhizoctonia solani* Kuhn at 0.25 mM with inhibition zone diameters of 11.57, 12.06, 8.5 and 10.21 mm, respectively. This suggested that **143** had broad inhibitory activity against these microbes [92]. **144** and **145** exhibited weak toxicity against brine shrimp (LC_50_ > 100 μM) and none of them displayed cytotoxicity against the liver hepatocellular carcinoma Huh7 and HepG2 (LC_50_ > 100 μM) and obvious inhibitory activities towards three marine-derived bacteria, *Bacillus stearothermophilus*, *Pseudoalteromonas nigrifaciens* and *Bacillus amyloliquefaciens*, and two common pathogens, *P. aeruginosa* and *S. aureus* [23].

Among the five diorcinols, only **146** showed apparent cytotoxicity against murine Ehrlich carcinoma cells and hemolytic activity against mouse erythrocytes. The significant hemolytic activity of **146** suggested that its cytotoxic activity against murine Ehrlich carcinoma cells was due to a membranolytic mechanism. It is well known that the heat shock protein 70 (HSP70) was frequently overexpressed in tumor cell lines as an ATP-dependent molecular chaperone and played a significant role in refolding misfolded proteins and promoting cell survival under stress [94]. Thus, compounds that could inhibit HSP70 had great potential in tumor therapy. **147** could decrease the expression of HSP70 in the Ehrlich carcinoma cells, which made it possible to develop as a new antitumor drug/lead. Diorcinol D (**149**) was studied for its combined therapy against planktonic *Candida albicans* with a broad-spectrum antifungal agent fluconazole [95]. The combined therapy exhibited considerable antifungal activity against ten clinical isolates of *C. albicans* containing five fluconazole-resistant isolates and five fluconazole-sensitive isolates, whereas fluconazole alone did not display antifungal activity. This suggested that diorcinol D (**149**) restored the susceptibility of fluconazole to *C. albicans*.

Moreover, the efficiencies of fluconazole inhibiting mature biofilms were also drastically boosted by the addition of **149** [95]. The fractional inhibitory concentration index (FICI) model and Δ*E* model unclosed that the synergistic actions indeed existed in combination of diorcinol D (**149**) and fluconazole [95]. Two resistance mechanisms of azoles were overexpression of efflux pumps genes and alterations of genes (point mutations). **149** mainly suppressed the activity of efflux pump in cells partly by decreasing the expression of Cdr1 (one mediator of azole efflux pumps) in *Candida albicans* CASA1. On the other hand, **149** also inhibited ergosterol synthesis and CYP51 (the target of fluconazole) expression [95]. Thus, the significant synergistic interaction and drug-resistant reversion of fluconazole combined with diorcinol D (**149**) were caused by the two latent mechanisms, the block of efflux pump and ergosterol biosynthesis. Notably, **149** was still needed to further in vivo study in the combination therapy field to settle rock-ribbed clinical fungal infection in response to the azole resistance.

#### 2.9.2. Other Compounds Derived from the Co-Cultures of Marine Fungi and Bacteria

**Figure 30:**
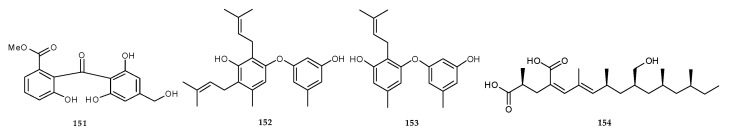
Chemical structures of **151**–**154**.

Five known metabolites, diorcinol D (**149**), penicillanone (**151**), diorcinol G (**152**), diorcinol I (**153**) and radiclonic acid (**154**) (Figure 30) were obtained from the co-culture of the sponge-derived fungi *A. versicolor* and *B. subtilis* [38].

Compounds **149**, **152** and **153** displayed antibacterial activities against five Gram-positive microbes, including one *S. aureus*, two *E. faecalis* and two *E. faecium* with the MIC values of 12.5–50 μM. In addition, **152** displayed potent inhibitory activities against all tested bacteria with an MIC value of 12.5 μM. **149** displayed inhibitory activity against *E. coli* with an MIC value of 8 µg/mL; and **153** showed significant antibacterial activity against *S. aureus* with an MIC value of 6.25 μg/mL [96,97]. In contrast, **149**, **152** and **153** did not display any obvious activity against L5178Y cell lines, which suggested that the antimicrobial activities of these products were not associated with their respective general toxicities [38].

## 3. Conclusions

Marine microorganisms have attracted more attention as natural producers of lead compounds. Marine microbes especially are considered as a renewable and reproducible source that can be easily cultured [98,99]. However, the speed of new lead compound discovery is slowing down. Thus, marine microbial co-culturing represents a powerful strategy for the production of novel bio-substances. The strategy can induce the biosynthesis of novel compounds and various NPs coded by corresponding genomes through the activation of the silent gene clusters or previously unexpressed biosynthetic routes.

In the last ten years, the overall statistical studies showed that 156 metabolites were discovered from the co-culture of different marine microbes. Figure 2 and Table 1 illustrated that 59 compounds were isolated from the co-culturing of different marine fungi; 79 compounds were isolated from marine fungi and bacteria; and only 18 compounds were disclosed from co-culturing different marine bacteria. The metabolites by co-culture of marine fungi and bacteria accounted for the largest proportion (51% of all metabolites of marine microbial co-culture). Alkaloids were the largest group with ≥51.9%, whereas macrolides were the lowest group with <0.65%. Just only one macrolide was identified from the co-cultures of different marine bacteria. Furthermore, co-cultures of different marine bacteria did not produce cyclopeptides, phenylpropanoids and steroids, and co-cultures of different marine fungi did not induce the biosynthesis of terpenoids.

Several studies suggest that *Aspergillus* spp. are the most common fungi that co-fermented with other microbes and produce numerous novel skeletons. The majority of these NPs have antimicrobial or/and antitumor activities. However, some significant restrictions obstruct the development of the co-culture technology; e.g., cryptic and undefined biosynthesis routes and the producers of NPs from the co-cultivation of two or more microorganisms, the particularities of strains and environmental and nutritional requirements, the instability of the ecological relationship, the uncertainty of the interaction relationship and the high contamination probability. Therefore, new technology and equipment need to be created, such as metabolomics analysis and molecular network technology. The new mechanisms of chemical communication of microbes (through direct/mediate contact) also need to be further investigated. In conclusion, co-culture is still shrouded in mystery as a prospective experimental tool for novel bioactive NPs. This article embodies the value and diversity of NPs from the co-cultivation of marine-derived microorganisms and it is considered as a guided reference for studying NPs.

## Figures and Tables

**Table 1:** Summarized NPs identified from the co-culture of marine microbes: 2009–2019.

Classes	The Number of NPs	Identified Date	Bioactivities	Co-Culture of Marine Microorganisms
Alkaloids	80 isolates (**1**–**80**)	2010 and 2014–2019	Cytotoxicity, enzyme Inhibitors, antimicrobial activities	**Fungi and fungi**
				*A. sulphureus* KMM 4640 and *I. felina* KMM 4639 *Aspergillus.* sp. FSY-01 and FSW-02 *P. citrinum* SCSGAF 0052 and *A. sclerotiorum* SCSGAF 0053 *Phomopsis* sp. K38 and *Alternaria* sp. E33
				**Fungi and bacteria**
				*Penicillium* sp. DT-F29 and *Bacillus* sp. B31 *A. flavipes* fungus and *S.* sp. CGMCC4.7185 *A. fumigatus* MR2012 and *S. leeuwenhoekii* C34 *A. versicolor* and *B. subtilis*,
				**Bacteria and bacteria**
				*Streptomyces* sp. CGMCC4.7185 and *B. mycoides* *Saccharomonospora* sp. UR22 and *Dietzia* sp. UR66
Anthraquinones	13 isolates (**81**–**93**)	2017–2019	Cytotoxicity and antimicrobial activities	**Fungi and fungi**
				Asexual morph and sclerotial morph of *A. alliaceus*
				**Fungi and bacteria**
				*A. versicolor* and *B. subtilis*
				**Bacteria and bacteria**
				*Micromonospora* sp. WMMB-235 and *Rhodococcus* sp. WMMA-185
Cyclopeptides	6 isolates (**94**–**99**)	2014 and 2019	Antifungal and anti-proliferative activities	**Fungi and fungi**
				*Phomopsis* sp. K38 and *Alternaria* sp. E33 *Aspergillus* sp. BM and 05-BM-05ML
				**Fungi and bacteria**
				*A. versicolor* and *B. subtilis*
Macrolides	1 isolate (**100**)	2018	Antitumor and antibacterial activity	**Bacteria and bacteria**
				*Saccharomonospora* sp. UR22 and *Dietzia* sp. UR66
Phenylpropanoids	23 isolates (**101**–**123**)	2011, 2015 and 2019	Cytotoxic, antifungal, antibacterial and anti-influenza activities	**Fungi and fungi**
				*Phomopsis* sp. K38 and *Alternaria* sp. E33 *A. sydowii* EN-534 and *P. citrinum* EN-535
				**Fungi and bacteria**
				*A. versicolor* and *B. subtilis*
Polyketides	12 isolates (**124**–**135**)	2013, 2014 and 2018	Anti-proliferative, cytotoxicity and antifungal activities	**Fungi and fungi**
				*Aspergillus* sp. BM and 05 and BM-05ML *Penicillium* sp. Ma(M3)V and *Trichoderma* sp. Gc(M2)1
				**Fungi and bacteria**
				*Penicillium* sp. WC-29-5 and *S. fradiae* 007
				**Bacteria and bacteria**
				*Janthinobacterium* spp. ZZ145 and ZZ148
Steroids	5 isolates (**136**–**140**)	2009, 2010 and 2014	Antiproliferative activity	**Fungi and fungi**
				*Aspergillus* sp. FSY-01 and FSW-02
				**Fungi and bacteria**
				*Aspergillus* sp. BM05 and an unknown bacteria (BM05BL)
Terpenoids	2 isolates (**141**–**142**)	2012 and 2017	Inhibition of diatom *N. annexa* and macroalga *U. pertusa*	**Fungi and bacteria**
				*A. fumigatus* MR2012 and *S. leeuwenhoekii* C58
				**Bacteria and bacteria**
				*S. cinnabarinus* PK209 and *Alteromonas* sp. KNS-16
Others	12 isolates (**143**–**154**)	2013, 2016, 2017 and 2019	Antimicrobial, toxicity, cytotoxicity, Hemolytic activities	**Fungi and fungi**
				*Phomopsis* sp. K38 and *Alternaria* sp. E33 *P. citrinum SCSGAF* 0052 and *A. sclerotiorum SCSGAF* 0053 *A. sulphureus* KMM 4640 and *I. felina* KMM 4639
				**Fungi and bacteria**
				*A. versicolor* and *B. subtilis*
